# Chinese Society of Clinical Oncology Breast Cancer (CSCO BC) guidelines in 2022: stratification and classification

**DOI:** 10.20892/j.issn.2095-3941.2022.0277

**Published:** 2022-06-29

**Authors:** Jianbin Li, Zefei Jiang

**Affiliations:** 1 Senior Department of Oncology, Fifth Medical Center of Chinese PLA General Hospital, Beijing 100071, China

In April 2017, the first edition Chinese Society of Clinical Oncology Breast Cancer (CSCO BC) guidelines were released. These guidelines were based on medical evidence, as well as the accessibility and cost effectiveness of various anticancer drugs in China. The CSCO BC guidelines include regimens with a high level of evidence, good product accessibility and high consistent consensus among Chinese experts as level I recommendations. Regimens with a high level of evidence but poor product accessibility or expert consensus are included as level II recommendations. Regimens that are clinically applicable but have a low evidence level are included as level III recommendations. The CSCO BC guidelines with these hierarchical recommendations have been widely accepted and are updated once annually according to the latest research progress in China and other countries.

In recent years, drug discovery focused on particular target genes in breast cancer has yielded various newly developed drugs, such as trastuzumab and CDK4/6 inhibitors. As more patients with early breast cancer have used these drugs, the regimens chosen in metastatic stages have been greatly affected by those chosen in early stages. Therefore, new stratification and classification strategies based on maximum benefit principles and previous treatments are highlighted in the CSCO BC guidelines. For example, patients with HER2 positive metastatic breast cancer can be divided into trastuzumab-sensitive and trastuzumab-resistant patients. Chemotherapy can also be divided into taxane-sensitive and taxane-resistant regimens. Stratifying these treatments according to key drugs is more reasonable than stratification by therapy stage. The 2022 CSCO BC guidelines have been updated according to the concept of classification and stratification treatment of breast cancer^1^. Here, we discuss the major updates included in the 2022 CSCO BC guidelines.

## Neoadjuvant therapy in HER2 positive breast cancer

In 2020, the inclusion of pertuzumab among treatments with medical insurance coverage solved the problem of poor treatment accessibility for patients with HER2 positive breast cancer in China. Trastuzumab and pertuzumab based regimens, such as TCHP and THP, have become basic choices for patients receiving neoadjuvant therapy. The KATHERINE^2^ study has led to changes in the patterns of adjuvant target therapy after neoadjuvant therapy. In this year, the CSCO BC guidelines first included a special chapter recommending adjuvant target therapy for patients after completion of neoadjuvant therapies. For patients achieving pathologic complete remission (pCR), the CSCO BC guidelines recommend continuation of the original targeted drugs in the neoadjuvant stage. However, if patients have prior use of only trastuzumab, dual target regimen is preferable in adjuvant therapy, according to the APHINITY study^3^.

**Figure 1:**
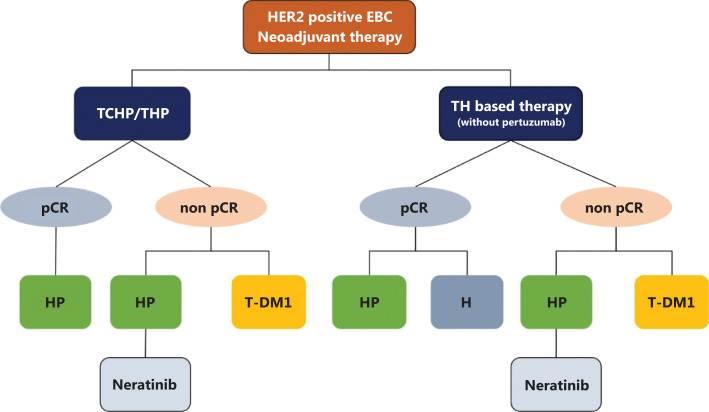
Recommendations for neoadjuvant and adjuvant therapy for HER2 positive breast cancer. pCR, pathological complete remission; TCHP, taxane, carboplatin, trastuzumab and pertuzumab; THP, taxane, trastuzumab and pertuzumab; HP, trastuzumab and pertuzumab; T-DM1, trastuzumab emtansine.

For patients who have achieved non-pCR after neoadjuvant therapy, trastuzumab emtansine (TDM1) or dual target therapy are recommended. However, the KATHERINE study, designed to compare the efficacy between trastuzumab and TDM1, has reported that less than 20% of patients received dual target therapy in a neoadjuvant setting. No available evidence has indicated whether dual target therapy or TDM1 is better. Most experts in China believe that optimized regimens should be chosen on the basis of pathological results^4^. In the CSCO BC guidelines, dual target therapy is recommended if tumor lesions significantly decrease (Miller and Payne grade 3 or 4, etc.). In contrast, for lesions showing less of a decrease (Miller and Payne grade 1 or 2, etc.), switching to trastuzumab emtansine is recommended. (**Figure 1**)

Given the positive results of the ExteNET study^5^, as well as its coverage by Chinese medical insurance in January of 2022, neratinib is recommended for patients who have completed adjuvant dual target therapy. However, neratinib after TDM1 is not recommended in the guidelines, because of the lack of evidence and the currently limited accessibility to TDM1.

## Adjuvant therapy for triple negative breast cancer (TNBC)

Chemotherapy is the major treatment for TNBC. Yet, with the development of new targeted drugs and the popularization of sequencing technology, more options have become available to patients with TNBC. The OLYMPIA study^6^ has shown that, for patients with BRCA mutations, olaparib after adjuvant chemotherapy can increase disease free survival. For these patients, the U.S. Food & Drug Administration (FDA) approved olaparib for adjuvant therapy in March 2022, thus making BRCA genotyping a routine procedure and consequently making TNBC classification based on BRCA genotype more reasonable.

**Figure 2:**
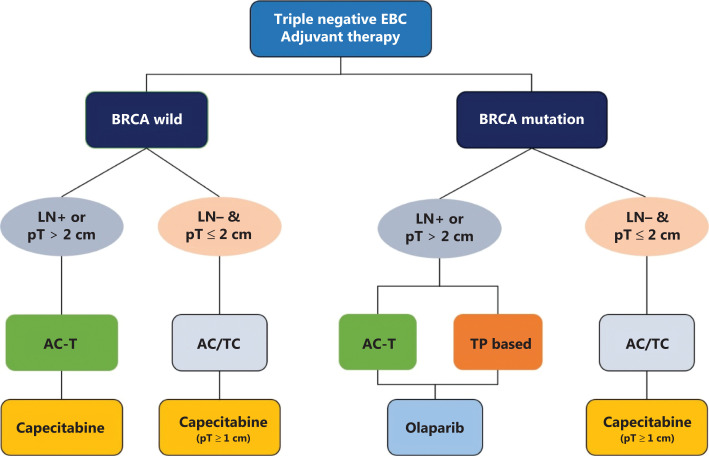
Recommendations for adjuvant therapy in triple negative breast cancer. LN+: lymph node positive; pT: pathologic tumor size. AC-T, anthracyclines, cyclophosphamide followed by taxane; AC, anthracyclines and cyclophosphamide; TC, taxane and cyclophosphamide; TP, taxane and platinum.

For patients with wild-type BRCA, adjuvant chemotherapy, such as anthracycline combined with cyclophosphamide followed by taxane (AC-T) or AC/TC, has been recommended according to patients’ risk factors (**Figure 2**). For patients with BRCA mutations, the guidelines suggest intensive chemotherapy because of the high recurrence risk. The PATTERN study^7^ has reported that for TNBC, particularly in young and high risk patients, there was a survival improvement with TP (paclitaxel combined with carboplatin) compared with FEC-T (cyclophosphamide, epirubicin, and fluorouracil followed by docetaxel). For patients with TNBC who have a young age or BRCA mutation, a platinum-based regimen is recommended in the CSCO BC guidelines.

For some patients with high-risk factors, a subsequent treatment after completion of the initial adjuvant chemotherapy is recommended by the guidelines. The SYSUCC-001 study^8^ has shown that for patients with TNBC, regardless of BRCA mutation status, capecitabine 1 year after routine chemotherapy can further decrease the recurrence risk. According to the inclusion criteria, the 2022 CSCO BC guidelines recommend capecitabine for 1 year for TNBC with tumors > 1 cm or lymph node metastasis. On the basis of the OLYMPIA study, olaparib is only a level II recommendation in the guidelines for patients with BRCA mutations, because of this drug’s unapproved indications and poor accessibility in China.

## Endocrine therapy for hormone receptor positive metastatic breast cancer

The stratification strategies between endocrine therapy and chemotherapy distinctly differ. For endocrine naïve patients, aromatase inhibitor (AI) is preferred. After failure of AI, fulvestrant is recommended. Moreover, studies have shown that the addition of targeted therapy can further improve the efficacy of endocrine therapy. On this basis, more attention should be paid to the classification of targeted drugs in choosing endocrine therapy.

The MONARCH plus study^9^ has confirmed that abemaciclib combined with AI or fulvestrant improves progression free survival (PFS), thus prompting the approval of abemaciclib in China. In 2018, AI combined with palbociclib was also approved for medical insurance coverage in China. The DAWNA-1 study^10^ has shown that, in addition to the above two CDK4/6 inhibitors, dalpiciclib combined with fulvestrant improves PFS. On this basis, this domestic CDK4/6 inhibitor was also approved and included in the CSCO BC guidelines.

Given the increase in the number of CDK4/6 inhibitors available in China, the CSCO BC guidelines provide different recommendations according to the accessibility and indications of different products. Abemaciclib combined with AI or fulvestrant is listed as a level I recommendation for patients who are endocrine drug naïve, or have had tamoxifen or AI failure. Although palbociclib combined with AI or fulvestrant, and dalpiciclib combined with fulvestrant are listed as level I recommendations, their level of evidence is lower than that of abemaciclib.

Moreover, the ACE study^11^ has demonstrated that steroid AI combined with chiadmide also improves the PFS in patients with prior tamoxifen or none-steroid AI failure. The CSCO BC guidelines include steroid AI combined with chiadmide, the first approved histone deacetylase inhibitor for breast cancer treatment, as a level I recommendation for patients who failed to tamoxifen or none steroid AI treatment.

**Figure 3:**
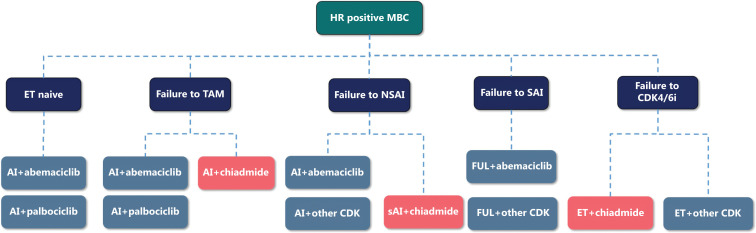
Recommendations for endocrine therapy in HR positive metastatic breast cancer. ET, endocrine therapy; TAM, tamoxifen; AI, aromatase inhibitor; NSAI, none steroid AI; SAI, steroid AI; FUL, fulvestrant; CDK, cyclin-dependent kinase 4/6 inhibitor.

CDK4/6 inhibitors combined with endocrine therapy have become the standard treatment for HR positive metastatic breast cancer. Notably, abemaciclib has also been an essential option for high-risk patients receiving adjuvant endocrine therapy since the publication of the MONARCHE study^12^. There is an increasing number of patients who fail to CDK4/6 inhibitors. However, limited data are available indicating possible preferred regimens in this population. Considering the potential evidence from clinical and real-world research, combined with suggestions from Chinese experts, the CSCO BC guidelines include chiadmide or another CDK4/6 inhibitor combined with endocrine therapy as level II recommendations. (**Figure 3**)

## Other updates

In 2022, the CSCO BC guidelines include several important new updates. For example, a new chapter has been added for adjuvant treatment after neoadjuvant treatment for TNBC. Hierarchical recommendations are made for rescue treatment of HER2 positive patients according to trastuzumab sensitivity, trastuzumab resistance, and tyrosine kinase inhibitor failure. Through this classification and stratification, we have been able to recommend the best treatment scheme for patients with breast cancer in different categories and stages.

The CSCO BC guidelines have regularly been updated according to the actual needs of patients. After the outbreak of COVID-19, we performed real world studies to explore the effects of the pandemic on the treatment of breast cancer^13^. Subsequently, suggestions for the management of patients with breast cancer during this pandemic have been proposed and included in the guidelines. Moreover, a new chapter concerning COVID-19 vaccination guidelines for patients with breast cancer has been added this year^14^, thereby providing an important reference for vaccination in various patient situations.

With technological development, artificial intelligent systems have also been widely used in clinical settings. We developed an artificial intelligence decision-making system based on the CSCO BC guidelines, named CSCO AI, to help clinicians quickly decide on the best regimens and improve decision-making efficiency^15^. The CSCO AI system has established a medical ecosystem integrating intelligent decision-making, toxicity warnings, patient management, and medical resource sharing functions. We believe that CSCO AI will help improve the standardized diagnosis and treatment of breast cancer in China.
